# The Y3** ncRNA promotes the 3′ end processing of histone mRNAs

**DOI:** 10.1101/gad.266486.115

**Published:** 2015-10-01

**Authors:** Marcel Köhn, Christian Ihling, Andrea Sinz, Knut Krohn, Stefan Hüttelmaier

**Affiliations:** 1 Institute of Molecular Medicine, Martin-Luther-University Halle-Wittenberg, Saxony-Anhalt 06120, Germany;; 2 Institute of Pharmacy, Martin-Luther-University Halle-Wittenberg, Saxony-Anhalt 06120, Germany;; 3 Interdisziplinäres Zentrum für Klinische Forschung, Core Unit DNA-Technologies, University Leipzig, Saxony 04103, Germany

**Keywords:** Y RNA, histone mRNA processing, Y3**, histone locus body, CPSF

## Abstract

We demonstrate that the Y3/Y3** noncoding RNAs (ncRNAs) bind to the CPSF (cleavage and polyadenylation specificity factor) and that Y3** associates with the 3′ untranslated region (UTR) of histone pre-mRNAs. The depletion of Y3** impairs the 3′ end processing of histone pre-mRNAs as well as the formation and protein dynamics of histone locus bodies (HLBs), the site of histone mRNA synthesis and processing. HLB morphology is also disturbed by knockdown of the CPSF but not the U7-snRNP components. In conclusion, we propose that the Y3** ncRNA promotes the 3′ end processing of histone pre-mRNAs by enhancing the recruitment of the CPSF to histone pre-mRNAs at HLBs.

## Abstract (precis)

In this study, Köhn et al. investigated how the cleavage and polyadenylation specificity factor (CPSF) is recruited to nascent histone pre-mRNAs during 3′ end processing of canonical histone mRNAs. They showed that the noncoding Y3/Y3** RNAs modulate the 3′ end processing of canonical histone mRNAs by binding to CPSF, thereby delineating a novel mechanism involved in the regulation of histone pre-mRNA processing.

Y RNAs are noncoding RNAs (ncRNAs) synthesized by RNA polymerase III in eukaryotes (Hall et al. 2013; Kohn et al. 2013; Wolin et al. 2013). Although their sequence is diversified, their structure and association with Ro60 are conserved (Sim and Wolin 2011). In mammals, four Y RNAs (Y1, Y3, Y4, and Y5) and isoforms (e.g., the Y3-derived Y3**) have been identified (Hendrick et al. 1981; Lerner et al. 1981; Wolin and Steitz 1983). Notably, Muroidea (mouse-like rodents) express only Y1 and Y3 but not Y3** (Wolin and Steitz 1983; Pruijn et al. 1993).

Y RNAs were suggested to modulate DNA replication and RNA quality control in association with RNA-binding proteins, in particular Ro60 (Christov et al. 2006; Sim and Wolin 2011; Kohn et al. 2013). Here, we identify the association of pre-mRNA processing factors such as the CPSF (cleavage and polyadenylation specificity factor) with Y1 and Y3/Y3**. The CPSF is essential for the 3′ end cleavage of pre-mRNAs (Shi et al. 2009; Chan et al. 2011). The 3′ end processing of most mRNAs relies on the polyadenylation signal (PAS) located 5′ to the cleavage site in mRNAs’ 3′ untranslated regions (UTRs) (Chan et al. 2011; Proudfoot 2011). Whereas the CPSF component WDR33 associates via the PAS, FIP1L1 binds flanking U-rich sequences (Kaufmann et al. 2004; Chan et al. 2014; Schonemann et al. 2014).

In contrast to the majority of mRNAs, replication-dependent histone mRNAs lack introns, and their 3′ end processing is PAS-independent. In their 3′ UTRs, the stem–loop (SL)-binding protein (SLBP) associates with a SL located 5′ to the histone downstream element (HDE). The latter binds to the U7-snRNP by an imperfect hybridization to the U7 ncRNA and is required for the 3′ end processing of histone mRNAs (Mowry and Steitz 1987). In addition, cleavage 3′ to the SL essentially relies on the CPSF CstF-64 (Yang et al. 2013) and is catalyzed by CPSF3 (Dominski et al. 2005). Correctly processed replication-dependent histone mRNAs lack a poly(A) tail, but aberrant polyadenylation occurs upon misprocessing (Dominski and Marzluff 2007; Marzluff et al. 2008; Pirngruber et al. 2009).

Histone genes are arranged in genomic clusters, forming discrete nuclear foci termed histone locus bodies (HLBs) (Shopland et al. 2001; Liu et al. 2006). NPAT (the key activator of histone mRNA synthesis) as well as 3′ end processing factors like Flash accumulate in HLBs (Bongiorno-Borbone et al. 2008; Ghule et al. 2008). How the CPSF is recruited to nascent histone pre-mRNAs in HLBs remained elusive.

Here, we demonstrate that the Y3** ncRNA promotes the processing of replication-dependent histone pre-mRNAs and modulates the morphology and protein dynamics at HLBs. Together with Y3**’s association with the CPSF and 3′ UTR of histone pre-mRNAs, this suggests that Y3** promotes the recruitment of the CPSF to nascent histone transcripts at HLBs.

## Results and Discussion

### Y RNAs associate with mRNA processing factors and promote the 3′ end processing of histone mRNA

**Figure 1.**
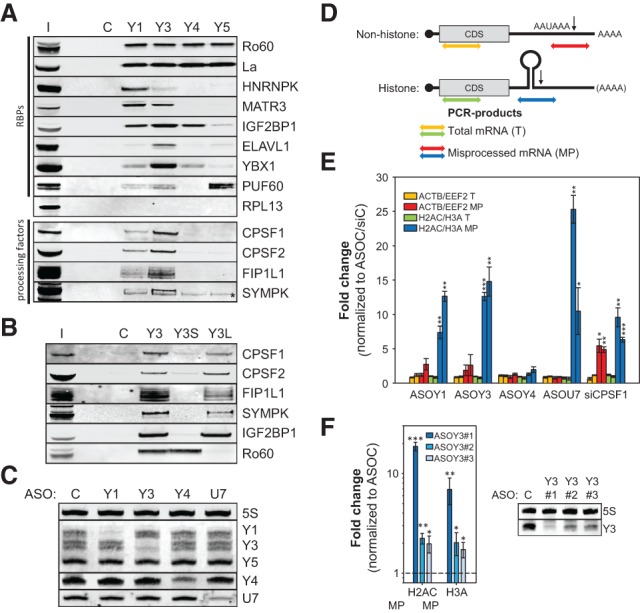
Y RNAs associate with processing factors and promote histone pre-mRNA processing. (*A*,*B*) RNA affinity purification with the indicated Y RNAs was performed in HEK293 cell lysates. Copurification of proteins (RBPs and processing factors) was determined by Western blotting with the indicated antibodies. (C) Bead control; (I) input fraction (5% of total); (*) cross-reactivity of anti-SYMPK. (*C*) HEK293 cells were transfected with control (C) or the indicated ncRNA-directed antisense oligonucleotides (ASOs) for 48 h. The depletion of ncRNAs was determined by infrared Northern blotting of total RNA with the indicated probes. 5S and Y5 ncRNAs served as loading controls. (*D*) Schematic of nonhistone and histone mRNAs, including the coding sequence (CDS) and the cleavage site (arrow), following the cleavage signals (PAS: AAUAAA or SL structure). Colored double arrows indicate the PCR products used for the quantification of (1) total nonhistone mRNAs (T; yellow), (2) misprocessed nonhistone mRNAs (MP; red), (3) total histone mRNAs (T; green), and (4) misprocessed histone mRNAs (MP; blue). (*E*) HEK293 cells were transfected with the indicated ASOs as in *C* as well as CPSF1-directed siRNAs. The levels of total (T) and misprocessed (MP) mRNAs (ACTB, EEF2, H2AC: HIST1H2AC; and H3A: HIST2H3A) were analyzed by quantitative RT–PCR (qRT–PCR) using R6 priming as depicted in *D*. The fold change of transcript levels was determined by the ΔΔC_T_ method relative to cells transfected with control ASOs/siRNAs using the PPIA-encoding mRNA for normalization. (*F*) HEK293 cells were transfected with control ASO (ASOC) or three distinct Y3-directed ASOs. The fold change of misprocessed H2AC and H3A mRNAs was determined as in *E*. The depletion of Y3 was monitored by Northern blotting as in *C*. Error bars indicate the SD of at least three independent analyses. Statistical significance was determined by Student's *t*-test, (*) *P* < 0.05; (**) *P* < 0.01; (***) *P* < 0.001.

To characterize the cellular role of human Y RNAs (Y1, Y3, Y4, and Y5), proteins associating with biotinylated Y RNAs in HEK293 cells were identified by mass spectrometry (Supplemental Fig. S1A–C; Supplemental Table S1). This revealed a partially Y RNA-specific enrichment of RNA-binding proteins (RBPs) and confirmed the association of Ro60 and La with all four human Y RNAs (Fig. 1A; Supplemental Fig. S1C). Although the previously reported association of the origin recognition complex (ORC) (Zhang et al. 2011) could not be confirmed (data not shown), the studies suggested that novel RBPs and, intriguingly, 3′ end processing factors associate with Y RNAs (Supplemental Fig. S1C). Western blotting confirmed the selective association of tested RBPs and binding of 3′ end processing factors to Y1 and Y3 (Fig. 1A). In Y3, these processing factors associated with a pyrimidine-rich (PR) stretch in the Y3 loop, as shown by truncated and chimeric ncRNAs (Fig. 1B; Supplemental Fig. S1D–F). IGF2BP1 served as the positive control for binding to the loop, and we could confirm a selective binding of Ro60 to the bulged Y RNA stem.

This copurification of processing factors suggested that Y1 and/or Y3 might modulate the 3′ end processing of mRNAs. This was analyzed by the RNase H-dependent knockdown of ncRNAs by chimeric antisense oligonucleotides (ASOs) as previously described (Ideue et al. 2009; Liang et al. 2011). Although we failed to deplete Y5, Northern blotting confirmed the efficient knockdown of Y1, Y3, Y4, and U7 ncRNAs (Fig. 1C). How Y RNA depletion affects the processing of nonhistone as well as histone mRNAs was initially analyzed by RT-qPCR by the indicated strategies (Fig. 1D). The knockdown of U7 and CPSF1 (depleted by siRNAs) served as controls for the misprocessing of selected histone (HIST1H2AC: H2AC; HIST2H3A: H3A) and nonhistone (ACTB and EEF2) mRNAs, respectively. As expected, the 3′ end processing of histone (H2AC and H3A) as well as nonhistone (ACTB and EEF2) mRNAs was significantly disturbed by the depletion of CPSF1 (Fig. 1E; Supplemental Fig. S1H). The knockdown of U7 resulted in a selective and severe up-regulation of misprocessed H2AC and H3A levels. Although the misprocessing of nonhistone mRNAs appeared modestly increased (not significant), the depletion of Y1 and Y3 but not Y4 significantly impaired the 3′ end processing of histone pre-mRNAs without affecting their total abundance (Fig. 1E). To test this in further detail, aberrant polyadenylation of the histone mRNAs H2AC and H3A was monitored by RT-qPCR (Supplemental Fig. S1G). As expected, aberrant polyadenylation was observed upon the depletion of U7 and CPSF1. Among analyzed Y RNAs, only the knockdown of Y1 and Y3 enhanced polyadenylation, providing further evidence for their role in the 3′ end processing of histone mRNAs. Since attempts to address Y RNA function by knockdown recovery studies failed, alternative Y3-directed ASOs were analyzed to reduce bias by off-target effects. Consistent with only moderate knockdown efficiencies, the additional ASOs only modestly but still significantly disturbed the processing of tested histone pre-mRNAs (Fig. 1F).

**Figure 2.**
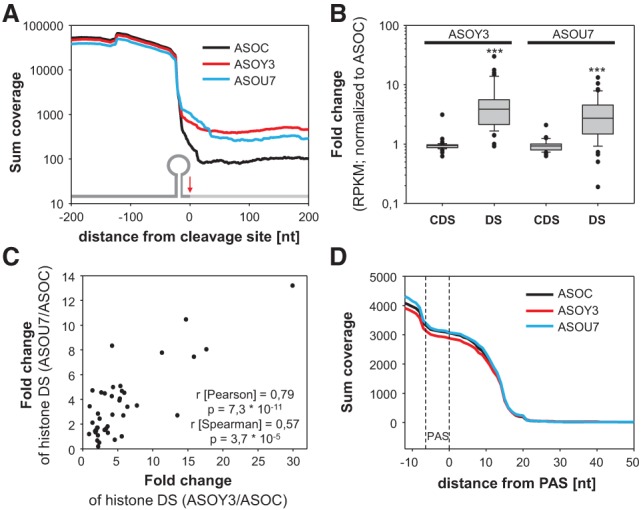
The depletion of the Y3 ncRNA impairs the 3′ end processing of replication-dependent histone pre-mRNAs. (*A*) Total RNA isolated from HEK293 cells transfected (48 h) with control (black), Y3-directed (red), or U7-directed (blue) ASOs was analyzed by RNA sequencing. The sum coverage of 46 histone transcripts observed in three independent analyses was determined 200 nucleotides upstream of and downstream from the cleavage sites. The inserted schematic indicates (5′ to 3′) (1) the SL, including the region 5′ of the cleavage site (dark gray); (2) the cleavage site (red arrow); and (3) the region 3′ of the cleavage site (light gray). (*B*) The fold change (to controls [ASOC]) of RPKM observed upon the depletion of Y3 or U7 in the CDS or 3′ of the cleavage site (DS) for each of the 46 histone transcripts is depicted in box plots. Statistical significance was determined by Student's *t*-test, (***) *P* < 0.001. (*C*) The fold change in DS abundance of U7-depleted samples was plotted over the respective change determined in Y3-depleted samples for each histone mRNA analyzed in *B*. Pearson and Spearman correlation parameters are indicated in the graph. (*D*) The sum coverage for 3′ ends of eight nonhistone mRNAs (ACTB, ACTG1, EEF2, GAPDH, RPL8, RPL29, RPS2, and PPIB) was analyzed as in *A*. The position of the PAS serving as the anchor is indicated by dashed lines.

Whether Y3 selectively and comprehensively modulates the 3′ end processing of replication-dependent histone mRNAs was analyzed by RNA sequencing. The sharp reduction of the sum coverage 3′ of the canonical cleavage sites of 46 replication-dependent histone mRNAs confirmed efficient processing in cells transfected with control ASOs (Fig. 2A, black). The depletion of U7 (Fig. 2A, blue) or Y3 (Fig. 2A, red) significantly elevated the sum coverage 3′ of cleavage sites, whereas the sum coverage in the 5′-flanking regions remained essentially unchanged. This indicated impaired 3′-cleavage without altered total abundance of replication-dependent histone pre-mRNAs. Transcript-dependent variations were analyzed by the RPKM (reads per kilobase per million)-normalized coverage in the coding sequence (CDS) and 3′ downstream region (DS) of all 46 histone transcripts (Fig. 2B). Despite some outliers, the depletion of Y3 or U7 significantly increased the DS reads, indicating deregulation of the vast majority of replication-dependent histone mRNAs. The quantitative assessment of misprocessing confirmed that processing is highly efficient, with only ∼0.4% of misprocessed transcripts in control cells (Supplemental Fig. S2A). Presumably due to incomplete depletion and pre-existing correctly processed transcripts included in the analyses, the knockdown of Y3 or U7 only led to moderately enhanced misprocessing (Y3: ∼2.3%; U7: ∼2.5%). Pearson as well as Spearman correlation analyses of fold changes in DS reads observed upon the depletion of Y3 or U7 confirmed that both ncRNAs modulate the 3′ end processing of histone pre-mRNAs in a comparable manner (Fig. 2C). Finally, the impact of Y3 and U7 depletion on the processing of eight nonhistone mRNAs (ACTB, ACTG1, EEF2, GAPDH, RPL8, RPL29, RPS2, and PPIB) was analyzed. Irrespective of ncRNA depletion, the sum coverage 3′ of the PAS dropped to zero (Fig. 2D). This indicated that the depletion of both ncRNAs did not affect the abundance or the 3′ end processing of the eight tested mRNAs. In conclusion, our studies revealed that Y1 and Y3 associate with 3′ end mRNA processing factors and selectively modulate the processing of replication-dependent histone mRNAs.

### The Y3** ncRNA promotes the 3′ end processing of histone pre-mRNAs

**Figure 3.**
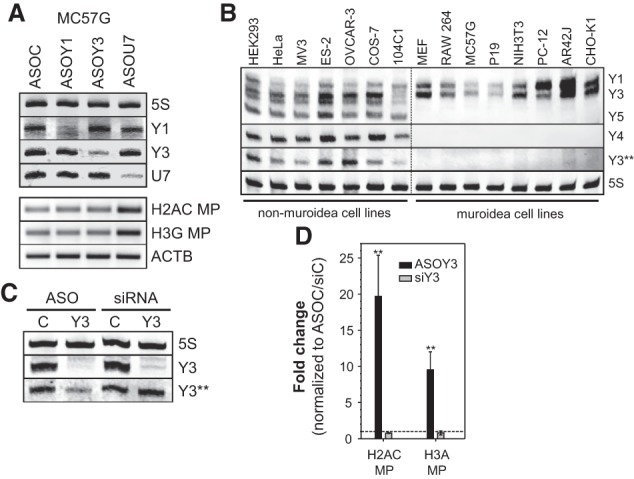
The Y3-derived Y3** ncRNA modulates the processing of human histone mRNAs. (*A*) Murine MC57G cells were transfected with the indicated ASOs for 48 h. (*Top* panel) RNA depletion was monitored by Northern blotting as in Figure 1C. (*Bottom* panel) Misprocessing of the indicated histone mRNAs and ACTB mRNA levels were determined by RT–PCR essentially as in Figure 1E. (*B*) Y RNA expression was analyzed in the indicated cell lines by Northern blotting as in Figure 1C. (*C*,*D*) HEK293 cells were transfected with sequence-identical control (C) or Y3-directed siRNAs or ASOs. (*C*) The depletion of Y3 and Y3** was monitored by Northern blotting as in Figure 1C. The 3′ end processing of the indicated histone mRNAs was analyzed by RT-qPCR as in Figure 1E. Error bars indicate the SD of at least three independent analyses. Statistical significance was determined by Student's *t*-test, (**) *P* < 0.01.

To test whether Y3's role in promoting the 3′ end processing of histone mRNAs is conserved in mammals, Y1 and Y3 were depleted in cells derived from mouse-like rodents (Muroidea). The 3′ end processing of two histone pre-mRNAs was monitored by RT–PCR. U7 served as a control due to its conserved role in the processing of histone pre-mRNAs (Fig. 3A; Supplemental Fig. S3A–C). As expected, the processing of histone pre-mRNAs was impaired by the knockdown of U7 in all analyzed cell lines. However, processing was unchanged by the depletion of Y1 or Y3 in all tested Muroidea-derived cell lines. For Y3, this was further analyzed in cells derived from non-Muroidea mammals (Supplemental Fig. S3C). In human-derived, monkey-derived, or guinea pig-derived cells, the depletion of Y3 and U7 led to an increased misprocessing of histone pre-mRNAs. This indicated that Y3's role is conserved in non-Muroidea cells and suggested that Y RNA-dependent regulation of DNA replication, previously reported in mouse-derived cells (Christov et al. 2006), was unlikely to involve their role in the processing of histone pre-mRNA. To test this further, the viability of human HEK293 and mouse B16-F10 cells was analyzed upon ASO-directed Y RNA and U7 depletion (Supplemental Fig. S3D,E). Although viability was significantly impaired by the depletion of Y1 or Y3 in both cell lines, it was barely reduced by the knockdown of Y4 or U7. In summary, these findings indicated that Y1/3's role in modulating cell viability is conserved, whereas they modulate the 3′ end processing of histone pre-mRNAs exclusively in non-Muroidea mammals. Northern blotting confirmed the expression of Y1 and Y3 and the lack of Y4 and Y5 in Muroidea-derived cells (Fig. 3B). We also noted that the Y3-dependent processing of histone pre-mRNAs was associated with the expression of Y3** in non-Muroidea (Wolin and Steitz 1983). As Y3** was depleted only by ASOs, whereas Y3 was depleted by sequence-identical ASOs and siRNAs in HEK293 cells (Fig. 3C), we analyzed whether these two depletion methods had different effects on histone RNA processing. RT–PCR analyses indeed confirmed that the ASO-directed knockdown of Y3/Y3**, but not the depletion of Y3 by siRNAs, impaired the processing of histone mRNAs (Fig. 3D). These findings indicated that Y3** rather than Y3 modulates the 3′ end processing of histone mRNAs in non-Muroidea and suggested that yet to be identified ncRNAs substitute for Y3** in Muroidea.

**Figure 4.**
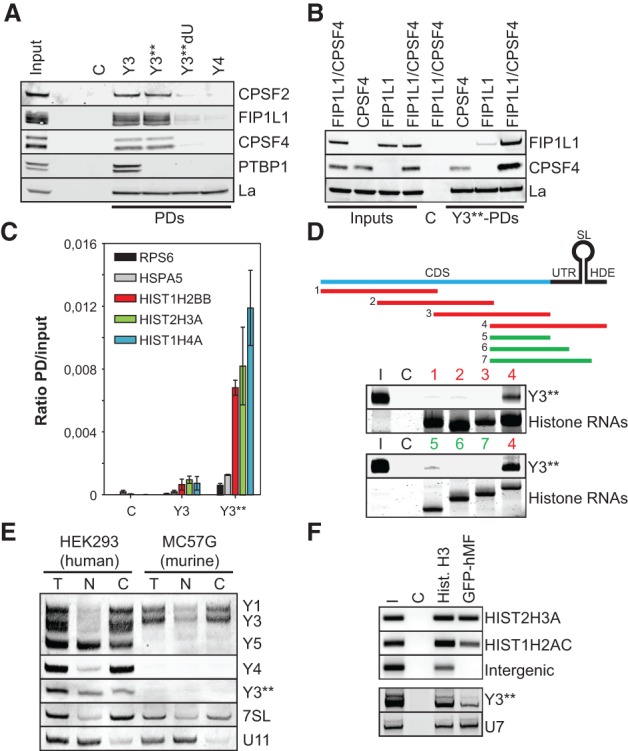
Y3** associates with the 3′ UTR of histone pre-mRNAs. (*A*) The copurification of proteins with the indicated Y RNAs, as in Figure 1C, was analyzed by Western blotting with the indicated antibodies. (C) Bead control; (I) input fraction (5% of total). (*B*) HEK293 cells were transfected with FIP1L1 and/or CPSF4 for 48 h as indicated. The pull-down (PD) of proteins with Y3**, as in Figure 1C, was analyzed by Western blotting. (C) Bead control; (I) input fractions (5% of total). (*C*) The copurification of the indicated mRNAs with biotinylated Y3 or Y3** was analyzed by RT-qPCR. The ratios of mRNA levels (pull-down/input) were determined by the ΔC_T_ method. Streptavidin resin served as a negative control (C). Error bars indicate the SD of at least three independent analyses. (*D*, *top* panel) Biotinylated H3A-derived transcripts used as bait for the copurification of Atto680-labeled Y3** in pull-down studies are depicted relative to the full-length H3A histone pre-mRNA using color coding. Copurification of Atto680-labeled Y3** and pull-down of biotinylated histone transcripts were monitored by infrared scanning (Y3**) or Syto60 staining (histone RNAs) of TBE-urea gels, respectively. Streptavidin resin (C) served as negative control. (I) Input (2% of total). (*E*) Subcellular localization of the indicated ncRNAs was analyzed by cell fractionation and Northern blotting of total (T), nuclear (N), or cytoplasmic (C) RNA isolated from human HEK293 or murine MC57G cells. U11 and 7SL served as controls for the enrichment of nuclear or cytoplasmic RNAs, respectively. (*F*) Chromatin immunoprecipitation (ChIP) analyses were performed in HEK293 stably expressing GFP-tagged human Mini-Flash (GFP-hMF). Beads only (C) served as a negative control, and Histone H3 served as a positive control. (*Top* panel) Eluted DNAs were PCR-amplified (HIST2H3A, HIST1H2AC, and intergenic DNA) and analyzed together with the input (I) on agarose gels. (*Bottom* panel) Coprecipitated RNAs were subjected to 3′-RACE, PCR-amplified, and analyzed by Southern blotting using the indicated probes.

To characterize the role of Y3** in further detail, its exact sequence had to be determined by 3′-RACE. This revealed termination at U60/61 of its precursor, Y3 (Supplemental Fig. S4A). Secondary structure predictions suggested that Y3** folds into a SL containing the U-rich stretch (PR) essential for CPSF binding in Y3 (Supplemental Fig. S4B). Consistently, RNA affinity purification confirmed the association of processing factors and La for Y3 and Y3**, whereas PTBP1 associated with only Y3, and Y4 bound only La (Fig. 4A). The deletion of the PR in Y3** (Y3**dU) abolished the association of processing factors. Notably, no association was observed for CSTF subunits, in particular CstF-64 (CSTF2), previously implicated in the processing of histone mRNAs (Supplemental Fig. S4C; Yang et al. 2013). Hence, despite partially distinct protein-binding properties, the CSTF-independent association of mRNA processing factors with Y3 was conserved in Y3**.

FIP1L1 and CPSF4 associate with U-rich sequences in pre-mRNAs (Barabino et al. 1997; Kaufmann et al. 2004), suggesting both factors as prime candidates for Y3/Y3** association. RNA affinity purification revealed that Y3** associated with both (Fig. 4B). Copurification was significantly enhanced when both factors were co-overexpressed, suggesting cooperative binding. Whether FIP1L1 directly associated with Y3** was analyzed by UV cross-linking of Atto680-labeled ncRNA and SBP-fused FIP1L1 in HEK293 cells (Supplemental Fig. S4D). Affinity purification of SBP-FIP1L1 confirmed direct binding to Atto680-Y3**, whereas no binding was observed for copurified CPSF4. Together, this suggested that Y3** associates with processing factors via the direct binding of FIP1L1 (Supplemental Fig. S4E).

An obvious way that Y3/Y3** could direct the CPSF complex to histone pre-mRNAs is an association with nascent histone transcripts. To test this, the association of two nonhistone mRNAs (RPS6 and HSPA5) and three histone transcripts (H2B, H3A, and H4A) with Y3 or Y3** was probed by RNA affinity purification (Fig. 4C). Strikingly, only the histone mRNAs significantly associated with Y3**, whereas no binding was observed for Y3. Consistently, Atto680-labeled Y3** was copurified with biotinylated H3A-derived transcripts comprising the HDE in RNA pull-down analyses (Fig. 4D). Notably, all attempts to detect an association of Y3** with histone transcripts in the absence of cell lysates (EMSA or upon psoralen cross-linking) failed (data not shown). Hence, although these findings cannot rule out that Y3** directly hybridizes to the HDE in histone pre-mRNAs, as proposed for U7, our findings rather support an indirect and protein-dependent association of Y3** with the HDE.

The proposed Y3**-directed recruitment of processing factors to nascent histone pre-mRNAs has to occur in the nucleus. Consistently, Northern blotting of fractionated (nucleus vs. cytoplasm) total RNA confirmed significant levels of Y3** in the nucleus of HEK293 cells (Fig. 4E). Y1, Y3, and Y4 were mainly cytoplasmic, whereas Y5 was enriched in the nucleus, supporting previous studies (Gendron et al. 2001). In murine MC57G lacking Y3**, subcellular sorting was confirmed for Y1 and Y3. Presumably due to their small size (limiting probe design), high sequence identity, and substantially higher levels of Y3 (∼100-fold) (Supplemental Fig. S4F), FISH analyses failed in confirming nuclear localization of Y3/Y3**. Therefore, we analyzed the recruitment of Y3** to HLBs, the proposed site of histone mRNA synthesis and processing. To this end, chromatin immunoprecipitation (CHIP) analyses were performed in HEK293 cells stably transduced with a GFP-tagged Mini-Flash (GFP-hMF), which localized to HLBs (Supplemental Fig. S5D), as previously reported (Burch et al. 2011). Consistent with its localization to HLBs, histone genes were readily copurified with GFP-hMF and histone H3, which served as a positive control (Fig. 4F, top panel). RNA association of GFP-hMF or histone H3 was analyzed by a 3′-RACE. U7 served as a positive control in these analyses. After PCR amplification of RACE products, Southern blotting confirmed the copurification of both Y3** and U7 ncRNAs, with GFP-hMF providing further evidence for a role of Y3** in the 3′ end processing of histone pre-mRNAs (Fig. 4F, bottom panel).

### Y3** modulates the morphology and protein dynamics of HLBs

**Figure 5.**
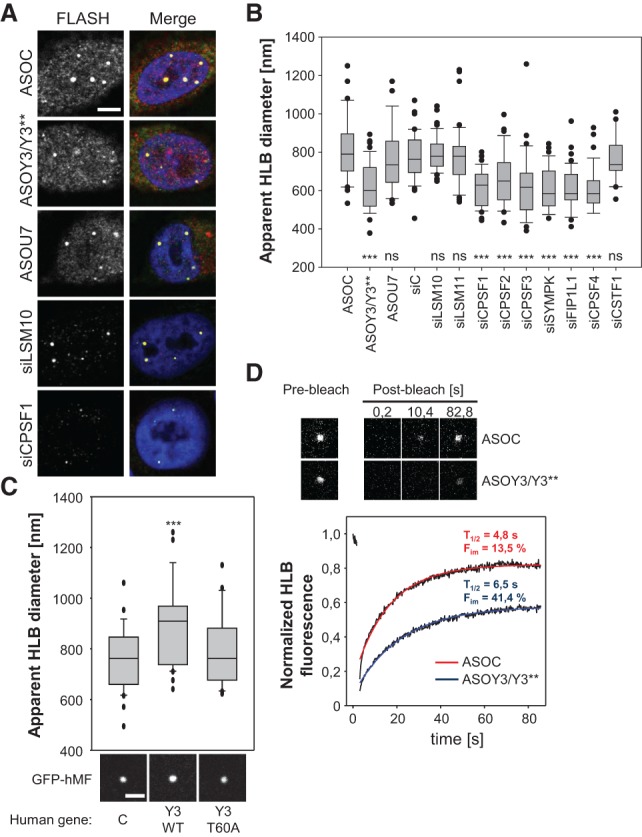
Y3** and the CPSF modulate HLB morphology and protein dynamics. (*A*,*B*) The morphology of HLBs was analyzed in human MV3 cells transfected with the indicated ASOs or siRNAs for 48 h by coimmunostaining of Flash and NPAT (see Supplemental Fig. S5A). (*A*) Representative Flash immunostaining and merged images (DAPI, Flash, and NPAT) are shown. Bar, 5 µm. (*B*) The apparent diameters of HLBs as determined by Flash staining and laser scanning microscopy is depicted by box plots for the indicated knockdowns. *n* > 35. (*C*) HLB morphology was analyzed in CHO-K1 cotransfected with GFP-hMF and empty vector (C) or vectors encoding human wild-type (Y3-WT) or T60A mutated (Y3-T60A) Y3 RNAs. (*Top* panel) The apparent HLB diameter was determined as in *B*. *n* > 35. Statistical significance was determined by Student's *t*-test, (***) *P* < 0.001. Representative images of GFP-hMF-positive HLBs are shown in the *bottom* panel. Bar, 3 µm. (*D*) HLB dynamics of GFP-hMF were analyzed by FRAP in HEK293 cells transfected with the indicated ASOs for 48 h. Representative images of HLBs before (prebleach) and at the indicated time points after photobleaching (post-bleach) are shown in the *top* panel. Averaged fluorescence recovery, including analyses of at least 17 HLBs per condition, were fitted and plotted in the *bottom* panel. Calculated half-time (*T*_1/2_) and immobile fraction (*F*_im_) are indicated.

The association of Y3** with HLB-localized GFP-hMF, mRNA processing factors, and histone pre-mRNAs suggested that the ncRNAs promote the recruitment of processing factors to HLBs and thus modulate HLB morphology and protein dynamics. The coimmunostaining of Flash and NPAT, two HLB-localized factors, revealed that the ASO-directed depletion of Y3/Y3** significantly reduced the apparent diameter of HLBs (Fig. 5A,B; Supplemental S5A,B). Surprisingly, this was not observed upon the knockdown of U7, suggesting that only the recruitment of Y3**-associated processing factors modulated HLB morphology. In agreement with this, the apparent diameter of HLBs was reduced by the siRNA-directed knockdown of CPSF-associated factors but remained unchanged by the depletion of U7-snRNP-associated LSM10/11 or CSTF1 that is not involved in the processing of histone pre-mRNAs (Fig. 5A,B; Supplemental Fig. S5A,B). The depletion of all analyzed processing factors was monitored by RT-qPCR (Supplemental Fig. S5C). Together, these findings suggested that the recruitment of Y3**-associated processing factors precedes the localization of the U7 snRNP at HLBs, a finding supporting the view that the recruitment of the U7-snRNP is a late event in HLB assembly (White et al. 2011). Since the knockdown of Y3** by ASOs also depletes Y3, we analyzed HLB morphology in cells lacking Y3**. To this end, CHO-K1 cells (hamster) were cotransfected with GFP-hMF to trace HLBs and wild-type (Y3-WT) or T60A-mutated (Y3-T60A) human *Y3* minigenes. The apparent diameters of HLBs were only increased in cells cotransfected with Y3-WT, which led to elevated expression of both Y3 and Y3** (Fig. 5C; Supplemental Fig. S5E). In contrast, HLB diameters remained largely unaffected by the cotransfection of Y3-T60A, which enhanced the expression of Y3 but not Y3**. This suggested that Y3** promotes the assembly of HLBs by recruiting processing factors to nascent histone transcripts, whereas Y3 is irrelevant for HLB assembly. We tested whether Y3** indeed modulates protein dynamics at HLBs using FRAP analyses in HEK293 cells stably expressing HLB-localized GFP-hMF (Fig. 5D). The ASO-directed depletion of Y3** severely prolonged the half-time and enhanced the immobile fraction of GFP-hMF in HLBs, providing further evidence that Y3** controls HLB assembly by promoting the recruitment of CPSF-associated processing factors.

In conclusion, we propose that the Y3** ncRNA promotes the recruitment of CPSF components to nascent replication-dependent histone pre-mRNAs at HLBs. These findings are largely consistent with previous studies showing that the Flash-dependent recruitment of processing factors guides the 3′ end processing of histone pre-mRNAs (Yang et al. 2013). However, our analyses suggest that the HLB recruitment of the CPSF by Y3** is independent of the U7-snRNP or the CstF64-containing histone pre-mRNA cleavage complex (HCC). Future studies will reveal the mechanisms of Y3** biogenesis, identify ncRNAs potentially substituting for Y3** in Muroidea, and address signaling events involved in these processes.

## Materials and methods

### Western and Northern blotting

Western and Northern blotting were essentially performed as previously described (Kohn et al. 2010, 2013). For antibodies and probes, see Supplemental Table S2. For ChIP analyses, see the Supplemental Material.

### RNA affinity purification

RNA affinity purification (60 pmol of RNA bait per sample) was performed as previously described (Wachter et al. 2013). For oligonucleotides used for the cloning of templates, see Supplemental Table S2.

### Cell culture and transfection

Cells were cultured in DMEM or RPMI 1640 supplemented with 1% GlutaMax and 10% FBS (Life Technologies). ASOs and siRNAs were transfected using RNAiMAX (Life Technologies). For each six-well plate (1.5 × 10^5^ to 5 × 10^5^ cells), 250 pmol of ASOs or siRNA was transfected for 48 or 72 h, respectively. For ASO/siRNA sequences, see Supplemental Table S2.

### Imaging

Indirect immunostaining was conducted as previously described (Stohr et al. 2006). Images were acquired using a Leica SP5x confocal microscope. Quantification of HLB sizes as well as FRAP analyses were performed using the Leica SP5 software. The apparent diameters of HLBs were determined by the Leica size quantification tool. FRAP analyses were performed with the Leica FRAP wizard.

### RNA sequencing, mass spectrometry, and RT–PCR

For RNA sequencing, mass spectrometry and RT–PCR analyses, see the Supplemental Material.

## Supplementary Material

Supplemental Material
