# Supplementary material for: The Y3** ncRNA promotes the 3′ end processing of histone mRNAs
Source: Genes Dev. 2015 Oct 1;29(19):1998–2003. doi: 10.1101/gad.266486.115 (PMC4604341; doi:10.1101/gad.266486.115)
Supplement: Supplemental Material [file supp_29_19_1998__index.html]

Supplemental Material 

# The Y3\*\* ncRNA promotes the 3′ end processing of histone mRNAs

## Supplemental Material

**Files in this Data Supplement:**

- Supp Figures.pdf
- Supp Material.docx
- Supp Table S1.xlsx
- Supp Table S2.xlsx
- Supp Table S3.xlsx
