# Supplementary material for: The Y3** ncRNA promotes the 3′ end processing of histone mRNAs
Source: Genes Dev. 2015 Oct 1;29(19):1998–2003. doi: 10.1101/gad.266486.115 (PMC4604341; doi:10.1101/gad.266486.115)

Köhn_Figure S1

A

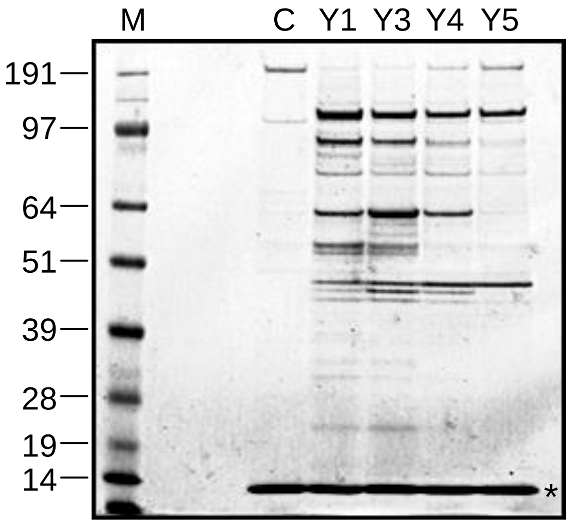

B

protein class hits 'nucleic acid binding'

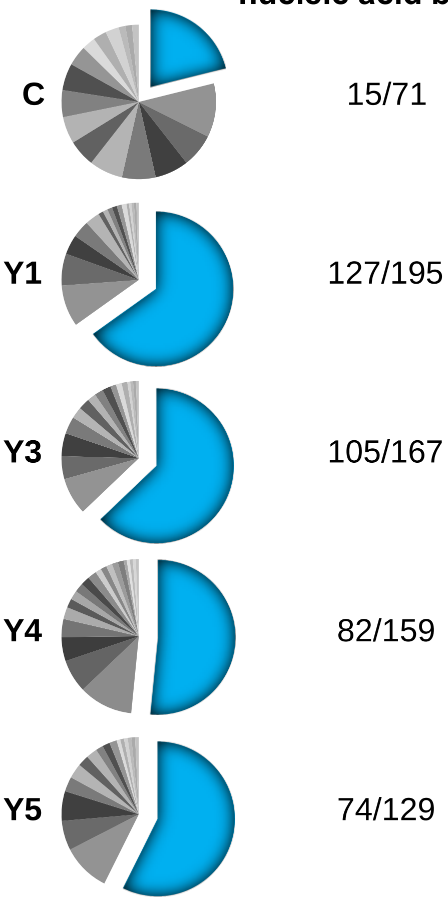

C

	Protein (Gene Symbol)	C	Y1	Y3	Y4	Y5
	Ro60 (TROVE2)		46,8<br>39	52,2<br>52	53,0<br>64	50,7<br>63
	La (SSB)	6,1<br>7	76,2<br>175	77,2<br>171	77,2<br>211	78,2<br>222
	HNRNPK		53,8<br>76	17,7<br>5	5,0<br>1	7,8<br>3
	MATR3	3,8<br>2	56,6<br>129	60,6<br>161		
	IGF2BP1	10,1<br>10	61,2<br>102	61,2<br>182	60,3<br>163	48,4<br>49
	ELAVL1		3,4<br>1	50,6<br>40	3,4<br>1	3,4<br>1
	YBX1		60,8<br>51	63,9<br>83	65,1<br>68	60,8<br>35
	PUF60	5,6<br>1	34,9<br>23	14,3<br>9	17,0<br>7	60,3<br>68
	RPL13		6,2<br>1	10,0<br>2		
3'-end processing factors	CPSF160 (CPSF1)		4,3<br>6	11,4<br>16		
3'-end processing factors	CPSF100 (CPSF2)		1,7<br>1	16,1<br>10		
3'-end processing factors	CPSF73 (CPSF3)			5,3<br>3		
3'-end processing factors	CPSF30 (CPSF4)			14,1<br>3		
3'-end processing factors	hFIP1 (FIP1L1)		2,9<br>1	14,8<br>7		
3'-end processing factors	WDR33			11,0<br>13		
3'-end processing factors	Symplekin (SYMPK)		2,2<br>2	4,1<br>4		

XX: Peptide coverage (PC) in % (blue; first value in each cell)

XX: Peptide spectral matches (PSMs) (red; second value in each cell)

Köhn_Figure S1

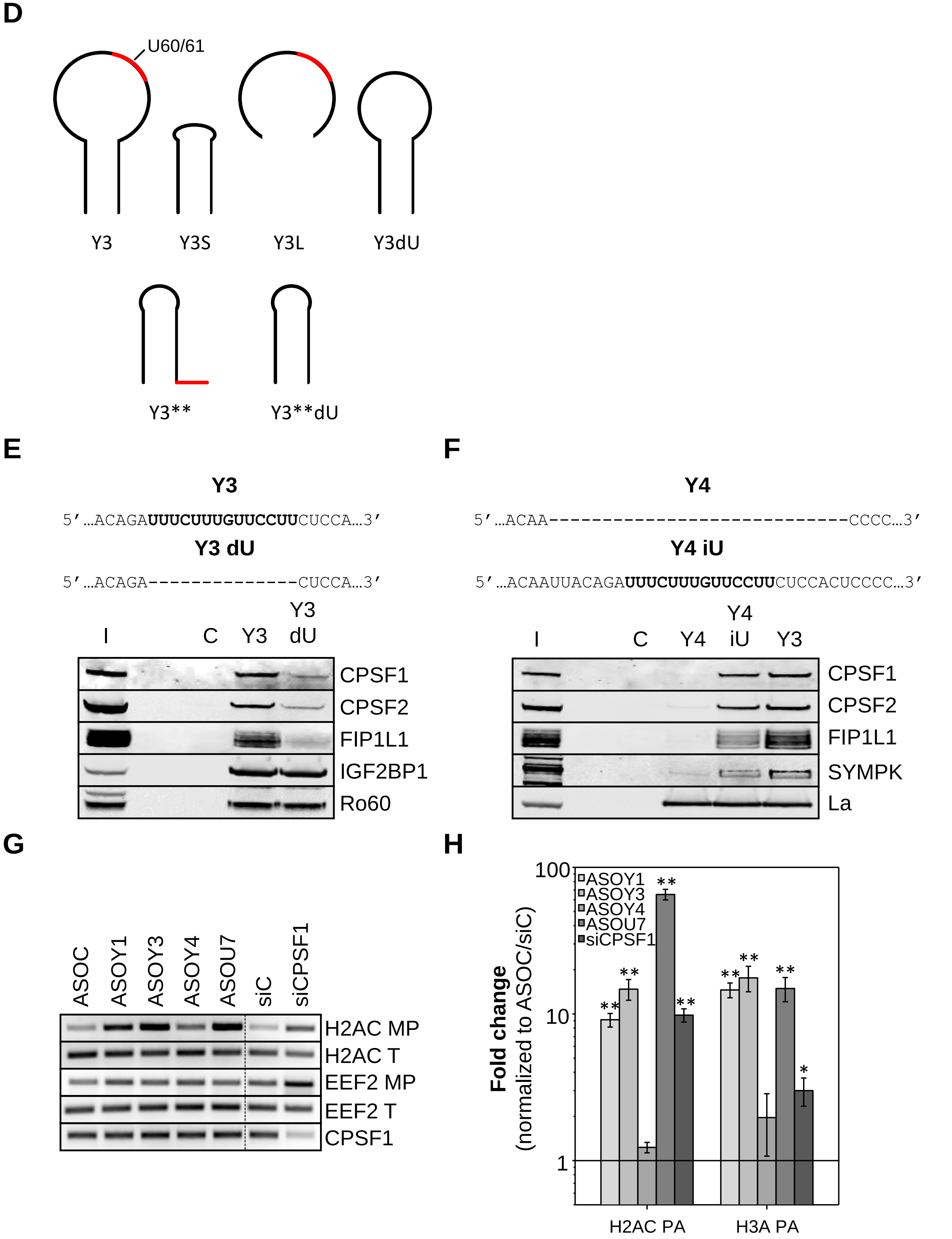

A

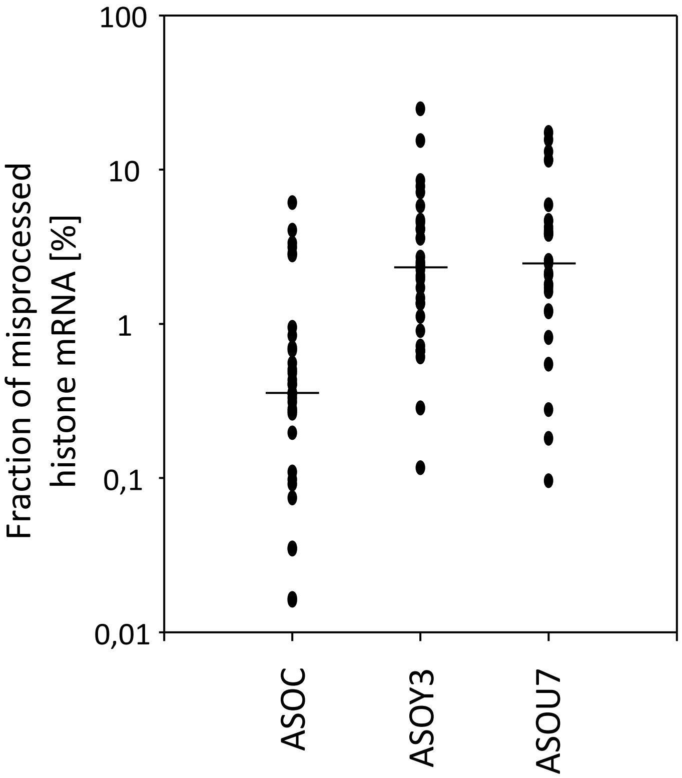
100
10
1
0,1
0,01
Fraction of misprocessed histone mRNA [%]
ASOC
ASOY3
ASOU7

Köhn_Figure S3

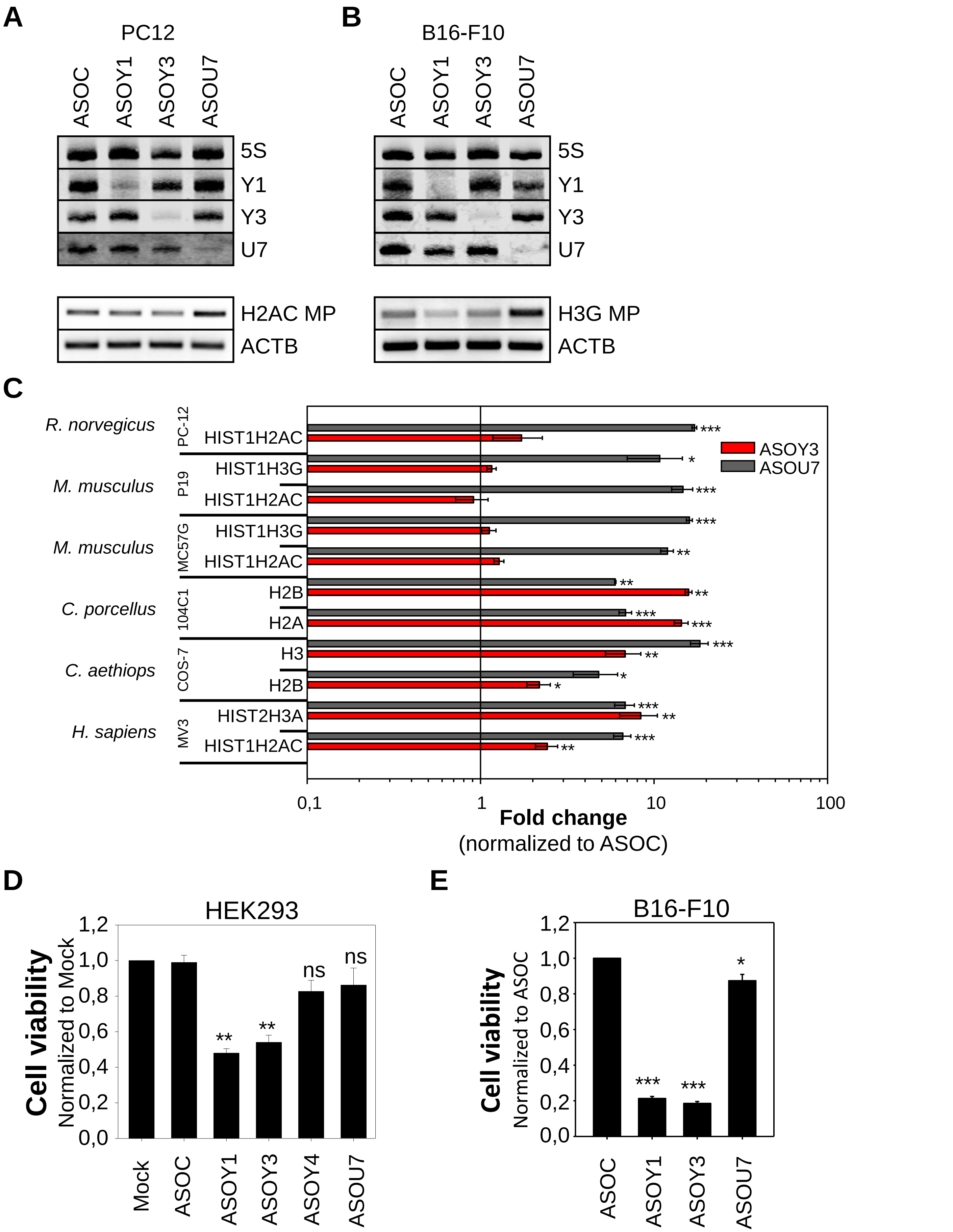

Köhn_Figure S4

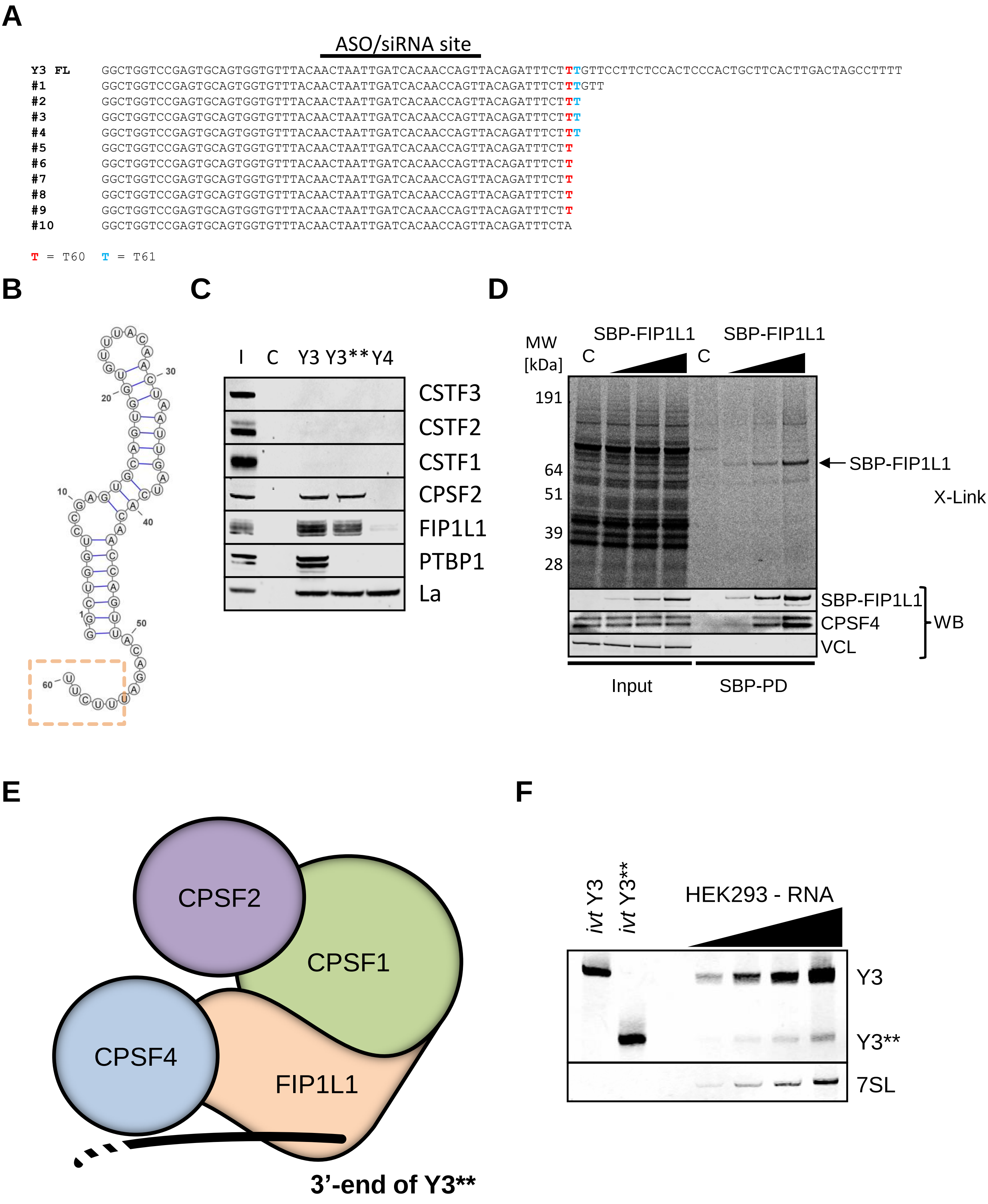

Köhn_Figure S5

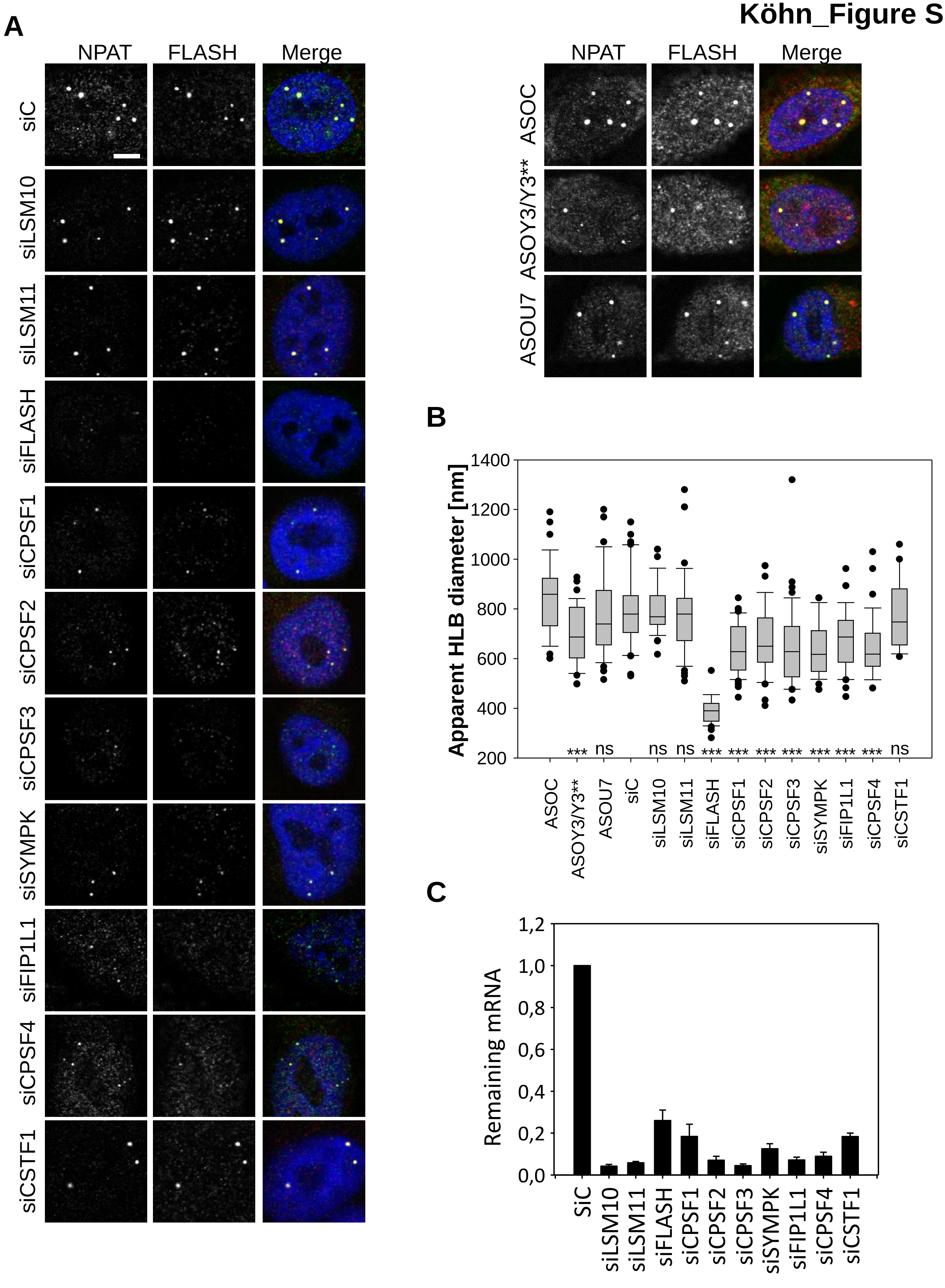

Köhn_Figure S5

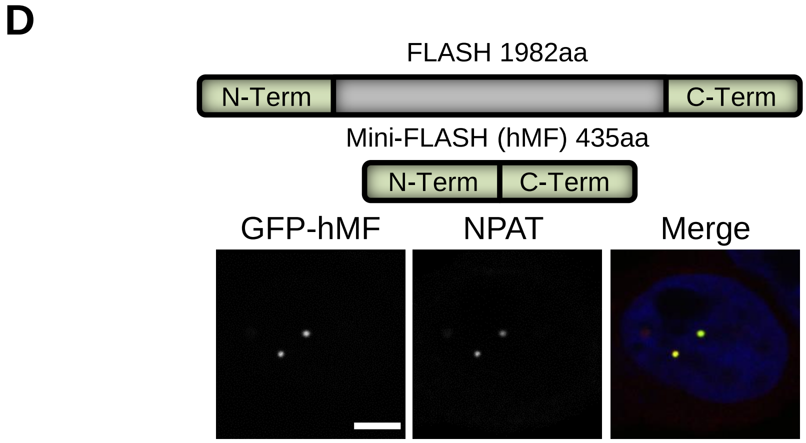

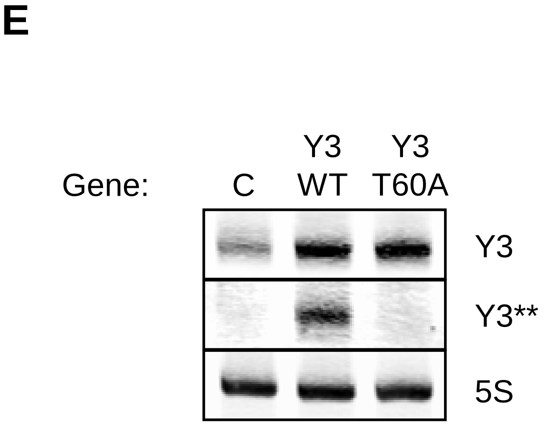

Supplement: Supplemental Material [file supp_29.19.1998_SuppFigures.pdf]
