# Supplementary material for: The Y3** ncRNA promotes the 3′ end processing of histone mRNAs
Source: Genes Dev. 2015 Oct 1;29(19):1998–2003. doi: 10.1101/gad.266486.115 (PMC4604341; doi:10.1101/gad.266486.115)
Supplement: Supplemental Material [file supp_29.19.1998_SuppMaterial.docx]

Supplemental Materials and Methods

### Chromatin immunoprecipitation (CHIP) and Southern blotting

CHIP assays were essentially performed as previously described ([Carey et al. 2009](#_ENREF_1)) with minor changes. To quench the crosslinker a 1 M Tris HCl (pH 7.4) solution was used. For cytoplasmic cell lysis binding buffer (10mM HEPES-KOH pH 7.2, 5mM MgCl_2_, 150mM KCl, 0.5% NP-40) was used. 200 µg of chromatin was used per IP, which was conducted with Dynabeads Protein G (Life Technologies). For primers used please refer to Table S2. For the 3’-RACE of the co-precipitated RNAs please refer to the respective supplemental material and methods section.

### Cloning

Molecular cloning was performed as described previously using primers listed in (Table S2, [Kohn et al. 2010](#_ENREF_3)). The cDNAs of FIP1L1, CPSF4 and Mini-FLASH were cloned by RT-PCR from HEK293 cDNA using specific primers and sub-cloned into appropriate target vectors (see Table S2). Y RNA mutants used for RNA affinity purification were generated by PCR using mutagenesis primers indicated in Table S2. The Y RNA genes were PCR amplified from HEK293-derived genomic DNA and cloned into pGEM-T vectors (Promega). All generated plasmids were verified by sequencing.

### Quantitative and Semi-quantitative RT-PCR

Total cellular RNAs were isolated by TRIZOL, as previously described ([Stohr et al. 2006](#_ENREF_8)). Reverse transcription was performed with M-MLV reverse transcriptase (Promega) according to manufacturer’s protocols, either using dT (15mer) or random hexamer (R6) priming. Subsequently the cDNAs were diluted with water and analyzed by SYBR Green based qRT-PCR using PCR Master Mix (Promega) and target specific primers (Table S2). Calculation of relative RNA levels was performed by the ΔΔC_t_-method ([Livak and Schmittgen 2001](#_ENREF_4)). Semi-quantitative PCRs were analyzed by 2 % (w/v) agarose gels stained with ethidium bromide.

### Determination of cell viability

Cell viability was analyzed with CellTiter-Blue assay (Promega) according to manufacturer’s protocols.

### UV Crosslinking

SBP-tagged FIP1L1 was purified from HEK293 cells lysates with streptavidin-resin (Life Technologies) after crosslinking in the presence of Atto680-labeled Y3** RNA, as previously described ([Kohn et al. 2010](#_ENREF_3)).

### 3’-RACE (rapid amplification of cDNA-ends)

Total cellular RNA isolated from HEK293 cells was separated on a 15 % TBE urea gel. After staining with ethidium bromide, RNAs migrating at ~60nt were excised and isolated. These RNAs were ligated to a 3’-adaptor (P-CGAGAUGGCGGCUUCCUGC-BIO) with T4 RNA ligase (Life Technologies) according to the manufacturer’s instructions. Afterwards cDNA synthesis was performed using the linker primer (gcaggaagccgccatctcg). The PCR on the resulting cDNA was performed with an Y3 specific forward primer (ggctggtccgagtgcagtgg) and the linker primer. PCR products were cloned into pGEM-T vector (Promega) and single clones were sequenced. PCRs on 3’-RACE cDNAs for Southern Blot were performed using specific forward and adaptor reverse primers. Southern blotting was essentially performed as described in the Northern Blotting section with minor changes. Native TBE gels (15 %) were used for electrophoresis. After transfer DNAs were denatured for 10 min with a 0.4 M NaOH solution.

### Preparation of RNA-libraries and RNA-sequencing

500 ng of total RNA was depleted of ribosomal RNA using the RiboMinus kit (Life Technologies) according to the instructions of the manufacturer. Depleted RNA was then fragmented by addition of 5x fragmentation buffer (200 mM Tris acetate, pH 8.2, 500 mM potassium acetate and 150 mM magnesium acetate) and heating at 94 °C for 3 min in a thermal cycler followed by ethanol precipitation with ammonium acetate and GlycoBlue (Life Technologies) as carrier. Fragmented RNA was then reversed transcribed using random hexamer and Superscript III (Life Technologies). The second strand was synthesized using the TargetAmp kit (Epicentre) according to the instructions of the manufacturer. The final steps of library preparation, e.g. blunt end repair, adapter ligation, adapter fill-in and amplification were done as previously reported ([Meyer and Kircher 2010](#_ENREF_5)). The barcoded libraries were purified and quantified using the Library Quantification Kit Illumina/Universal (KAPA Biosystems) according to the instructions of the manufacturer. A pool of up to 10 libraries was used for cluster generation at a concentration of 10 nM using an Illumina cBot. Sequencing of 2x100 bp was performed with an Illumina HiScan-SQ sequencer at the sequencing core facility of the IZKF Leipzig (Faculty of Medicine, University Leipzig) using third version chemistry and flowcell according to the instructions of the manufacturer. Demultiplexing of raw reads, adapter trimming and quality filtering was done as previously reported ([Stokowy et al. 2014](#_ENREF_9)). Lasergene (DNASTAR) was used for annotation and RPKM normalization. Coverages were determined by SeqMonk (Babraham Bioinformatics). For alignment details please refer to Table S3.

### Mass spectrometry

For protein identification each gel lane was cut into ten bands, the proteins were in-gel digested following standard protocols ([Shevchenko et al. 2006](#_ENREF_7)). Briefly, protein disulfides were reduced with DTT and cysteines were alkylated with iodoacetamide. Peptides extracts were analyzed by LC/MS on an Ultimate 3000 Nano-HPLC system (Dionex) coupled to an LTQ-Orbitrap XL mass spectrometer (Thermo Fisher Scientific) equipped with a nano-electrospray ionization source (Proxeon). The samples were loaded onto a trapping column (Acclaim PepMap C18, 100 μm x 20 mm, 5 μm, 100Å, LC Packings, Amsterdam, NL) and washed for 15 min with 0.1 % trifluoroacetic acid (TFA) at a flow rate of 20 μl/min. Trapped peptides were eluted with the separation column (Acclaim PepMap C18, 75 μm x 250 mm, 3 μm, 100Å, LC Packings), which had been equilibrated with 100 % A (5 % acetonitrile, 0.1 % formic acid). Peptides were separated with a linear gradient: 0-40 % B (80 % acetonitrile, 0.08 % formic acid) in 30 min, 40-100 % B in 10 min, 100 % B for 10 min. The column was kept at 30°C and the flow rate was 300 nl/min. During gradient elution, online MS data were acquired in data-dependent MS/MS mode: each high-resolution full scan (m/z 350 to 2000, resolution 60,000) in the orbitrap analyzer was followed by five collision-induced dissociation product ion scans in the linear trap for the five most intense signals in the full-scan mass spectrum (isolation width 2 Th, normalized collision energy 35). Data analysis was performed using the Proteome Discoverer 1.2 (Thermo Fisher Scientific). MS/MS data of precursor ions in the mass range 600-6000 were searched against the Swissprot Database (version 11/2011, tax. human, 20253 entries) using Mascot (version 2.3, Matrixscience). Mass accuracy was set to 3 ppm and 0.8 Da for precursor and fragment ions, respectively, carbamidomethylation of cysteines was set as fixed modification and oxidation of methionine as possible modification, two missed cleavages of trypsin were allowed.

# Supplemental Information for Köhn et al.

## Supplemental Figure Legends

### Figure S1. MS-analyses identify the association of processing factors with Y RNAs

(A) Coomassie staining of proteins co-purifying with biotinylated *in vitro* transcribed Y RNAs from HEK293 cell lysates. Streptavidin resin alone served as negative control (C). ‘*’: Streptavidin protein. Molecular weights (kDa) are indicated on the left.

(B) The pie charts indicate the fraction of MS-identified proteins classified as ‘nucleic acid binding’ by PANTHER analyses in the indicated Y RNA pulldown fractions (see Figure 1; [Mi et al. 2005](#_ENREF_6)). Only proteins with peptide coverage greater than 10% were included. Streptavidin resin without RNA served as negative control (C).

(C) Peptide-coverage in percent (PC) and peptide spectral matches (PSMs) of proteins probed for Y RNA-binding by RNA affinity purification in Figure 1A (MS-analyses in Y RNA pulldown fractions, see Table S1). Note that for four of the identified proteins (CPSF1, CPSF2, FIP1L1 and SYMPK) selective association with Y1 and Y3 was validated by RNA affinity purification and Western blotting in Figure 1A. 3’-end processing factors are indicated on the left.

(D) Schematic of the Y3/Y3**-variants used in this study. The pyrimidine-rich stretch within the Y3-loop is colored in red. Note that the position of U60/61, the proposed cleavage site for the production of Y3**, is indicated in the Y3-loop.

(E, F) Co-purification of proteins with biotinylated *in vitro* transcribed ncRNAs from HEK293 cell lysates was analyzed as in Figure 1A. The Ro60 and La proteins served as positive controls for the association with the stem of Y RNAs. Y3: full length; Y3S: stem-only; Y3L: loop-only; Y3dU: lacking the U-rich stretch (PR) in the loop; Y4: full length; Y4iU: Y4 with an insertion of Y3’s PR. Schematics in top panels indicate the deleted PR in Y3dU (E) and Y3’s PR inserted in Y4 (F). For further detail see Figure S1D.

(G) Samples from Figure 1E were subjected to semi-quantitative RT-PCR analyses using R6-priming. For non-histone mRNA processing EEF2 (T and MP) and for histone mRNA processing H2AC (T and MP) was analyzed. CPSF1 depletion was monitored with respective primers (see also Table S2).

(H) Samples from Figure 1E were analyzed by dT-priming and qRT-PCR to determine the amount of misprocessed and polyadenylated histone mRNAs. The same PCR strategy used in Figure 1E was applied here. Error bars indicate s.d. of at least three independent analyses. Statistical significance was determined by student's t-test (*p<0.05, **p<0.01).

### Figure S2. Y3 depletion alters histone mRNA processing at ‘transcriptome wide’ scale

(A) The coverage of histone mRNAs was calculated from the RNA seq experiments used in Figure 2. Therefore the coverage was determined right before and after the cleavage site of the respective histone mRNAs. Then a coverage ratio was calculated to yield the fraction of misprocessed histone mRNAs upon ASOC, ASOY3 or ASOU7 knockdown conditions. Note that for this analysis just histone mRNAs were used, which retrieved sequence reads after the cleavage site in control conditions (ASOC; n=33).

### Figure S3. Histone mRNA processing is not affected by depletion of Y3 in cells lacking Y3**

(A, B) PC12 (rat) and B16-F10 (mouse) cells were transfected with control, Y1, Y3 and U7 directed ASOs. As described in Figure 3A samples were subjected to Northern Blot as well as semi-quantitative RT-PCR analyses. 5S rRNA served as loading control for Northern Blots and ACTB mRNA for RT-PCRs, respectively.

(C) The cell lines MV3, COS-7, 104C1, MC57G, P19 or PC-12, derived from indicated species (left panel), were transfected with control (ASOC), Y3- (red) or U7-directed (grey) ASOs. The fold change in misprocessing of indicated histone transcripts was monitored by qRT-PCR relative to ASOC-transfected controls, as described in Figure 1D. The depletion of ncRNAs was monitored by Northern blotting, as described in Figure 1C (data not shown). Error bars indicate s.d. of at least three independent analyses. Statistical significance was determined by student's t-test (*p<0.05, **p<0.01, ***p<0.001).

(D, E) Cell viability of ASO transfectants (HEK293 and B16-F10) was determined by Cell titer Blue assay 48h after transfection. Error bars indicate s.d. of three independent analyses. Statistical significance was determined by student's t-test (ns - not significant, *p<0.05, **p<0.01, ***p<0.001).

### Figure S4. Y3** associates with histone mRNA processing factors

(A) The 3’-end of human Y3** was identified by rapid amplification of cDNA-ends (RACE) using total RNA isolated from HEK293 cells for reverse-transcription. Ten clones obtained by RACE were sequenced. The sequence of analyzed clones is shown in alignment to the human full length Y3 RNA revealing that most Y3** transcripts apparently end in U59-60 di- or U59-61 trinucleotide, essentially as previously suggested ([Wolin and Steitz 1983](#_ENREF_10)). The site where the ASO/siRNA used in this study targets Y3/Y3** is indicated.

(B) The depicted secondary structure of Y3** was *in silico* predicted by RNA fold (University of Vienna) and VARNA ([Darty et al. 2009](#_ENREF_2)). Note, that studies presented in Figure 4A suggest that the extending U-rich stretch (box) at the Y3**’s 3’-end is essential for the association of CPSF (also see Figures S1E and S1F).

(C) RNA pulldown analyses were performed as described in Figure 4A with indicated ncRNA baits. Western Blots of eluates and input (I) were probed with antibodies raised against components of the CstF-complex (CSTF1, CSTF2 and CSTF3). Beads only (C) served as negative control.

(D) Empty vector (C) and increasing amounts of SBP-tagged FIP1L1 were transfected into HEK293 cells. Cell lysates from transfectants were incubated with Atto680-labeled Y3** RNA and subjected to UV-Crosslinking. Lysates were then digested with RNAse A/T1 and subjected to SBP-pulldowns using streptavidin-resin. Inputs as well as pulldown samples were resolved on a SDS-Gel, followed by crosslink-detection using infrared scanning. Proteins were detected by Western Blotting using the indicated antibodies.

(E) Schematic of the proposed complex formation of indicated CPSFs and FIP1L1 (colored) with the Y3** RNA (black), as suggested by Figures 4 and S4. We propose that CPSF4 and FIP1L1 form a core complex with FIP1L1 directly binding the Y3** 3’-end which serves as a binding scaffold for CPSF1 and 2.

(F) Increasing amounts of total RNA isolated from HEK293 cells was subjected to infrared Northern blotting using probes hybridizing to Y3 and Y3**, as described in Figure 1C. *In vitro* transcribed (*ivt*) Y3/Y3** ncRNAs were used as references and 7SL as loading control. Note that Y3** is substantially less abundant (10-100fold) than its precursor Y3.

### Figure S5. Analyses of HLB morphology and protein dynamics

(A) MV3 cells were transfected with control (siC, ASOC) or siRNAs/ASOs directed against indicated factors. HLBs were analyzed by immunostaining of NPAT and FLASH, as described in Figure 5A. Merged images with nuclear staining by DAPI are shown in right panels. Bar, 5µm. For quantification of apparent HLB diameters please refer to Figure 5B and S5B.

(B) HLB diameters were quantified for knockdown conditions from Figure S5A as described in Figure 5B for NPAT-stainings.

(C) Knockdown efficiencies for respective processing factors used in Figures 5A and S5A was determined by qRT-PCR. PPIA and EEF2 mRNA served as normalization controls. Error bars indicate s.d. of three independent analyses.

(D) A schematic of the FLASH protein and the Mini-FLASH (hMF) reporter derived by deletion of the central domain is depicted in the upper panel. The localization of stably expressed GFP-Mini-FLASH (GFP-hMF) in the HLBs of HEK293 cells counterstained for NPAT and DAPI is shown in the lower panel. Bar, 6µm.

(E) Expression of ncRNAs in CHO-K1 cells transfected as in Figure 5C was analyzed by infrared Northern blotting, as described in Figure 1C. 5S rRNA served as loading control. Consistent with studies in other *muroidea*-derived cells, no endogenous Y3** is observed in CHO-K1 and Y3** cannot be processed from Y3-T60A.

### Table S1. Proteins co-purifying with Y RNAs from HEK293 cells

Proteins identified by MS-analyses are indicated with peptide coverage in percent and peptide spectral matches (PSMs) determined in the indicated Y RNA pulldown fractions (see Figure 1).

### Table S2. Summary of oligonucleotides and antibodies

Sequence information of ASOs, siRNAs, oligonucleotides and probes used for Northern blotting as well as antibodies used for Western blotting.

### Table S3. RNA-seq statistics

The alignment statistics of all nine RNA-seq samples (3xASOC, 3xASOY3 and 3xASOU7) are listed.

# Supplemental References

Carey MF, Peterson CL, Smale ST. 2009. Chromatin immunoprecipitation (ChIP). *Cold Spring Harb Protoc* **2009**: pdb prot5279.

Darty K, Denise A, Ponty Y. 2009. VARNA: Interactive drawing and editing of the RNA secondary structure. *Bioinformatics* **25**: 1974-1975.

Kohn M, Lederer M, Wachter K, Huttelmaier S. 2010. Near-infrared (NIR) dye-labeled RNAs identify binding of ZBP1 to the noncoding Y3-RNA. *RNA* **16**: 1420-1428.

Livak KJ, Schmittgen TD. 2001. Analysis of relative gene expression data using real-time quantitative PCR and the 2(-Delta Delta C(T)) Method. *Methods* **25**: 402-408.

Meyer M, Kircher M. 2010. Illumina sequencing library preparation for highly multiplexed target capture and sequencing. *Cold Spring Harb Protoc* **2010**: pdb prot5448.

Mi H, Lazareva-Ulitsky B, Loo R, Kejariwal A, Vandergriff J, Rabkin S, Guo N, Muruganujan A, Doremieux O, Campbell MJ et al. 2005. The PANTHER database of protein families, subfamilies, functions and pathways. *Nucleic Acids Res* **33**: D284-288.

Shevchenko A, Tomas H, Havlis J, Olsen JV, Mann M. 2006. In-gel digestion for mass spectrometric characterization of proteins and proteomes. *Nat Protoc* **1**: 2856-2860.

Stohr N, Lederer M, Reinke C, Meyer S, Hatzfeld M, Singer RH, Huttelmaier S. 2006. ZBP1 regulates mRNA stability during cellular stress. *J Cell Biol* **175**: 527-534.

Stokowy T, Eszlinger M, Swierniak M, Fujarewicz K, Jarzab B, Paschke R, Krohn K. 2014. Analysis options for high-throughput sequencing in miRNA expression profiling. *BMC Res Notes* **7**: 144.

Wolin SL, Steitz JA. 1983. Genes for two small cytoplasmic Ro RNAs are adjacent and appear to be single-copy in the human genome. *Cell* **32**: 735-744.
